# Long Noncoding RNA and Protein Interactions: From Experimental Results to Computational Models Based on Network Methods

**DOI:** 10.3390/ijms20061284

**Published:** 2019-03-14

**Authors:** Hui Zhang, Yanchun Liang, Siyu Han, Cheng Peng, Ying Li

**Affiliations:** 1 College of Computer Science and Technology, Key Laboratory of Symbol Computation and Knowledge Engineering of Ministry of Education, Jilin University, Changchun 130012, China; huizhang16@mails.jlu.edu.cn (H.Z.); ycliang@jlu.edu.cn (Y.L.); hansy15@mails.jlu.edu.cn (S.H.); chengpengeace@gmail.com (C.P.); 2 Zhuhai Laboratory of Key Laboratory of Symbol Computation and Knowledge Engineering of Ministry of Education, Zhuhai College of Jilin University, Zhuhai 519041, China

**Keywords:** lncRNA–protein interaction prediction, computational model, biological network science, machine learning

## Abstract

Non-coding RNAs with a length of more than 200 nucleotides are long non-coding RNAs (lncRNAs), which have gained tremendous attention in recent decades. Many studies have confirmed that lncRNAs have important influence in post-transcriptional gene regulation; for example, lncRNAs affect the stability and translation of splicing factor proteins. The mutations and malfunctions of lncRNAs are closely related to human disorders. As lncRNAs interact with a variety of proteins, predicting the interaction between lncRNAs and proteins is a significant way to depth exploration functions and enrich annotations of lncRNAs. Experimental approaches for lncRNA–protein interactions are expensive and time-consuming. Computational approaches to predict lncRNA–protein interactions can be grouped into two broad categories. The first category is based on sequence, structural information and physicochemical property. The second category is based on network method through fusing heterogeneous data to construct lncRNA related heterogeneous network. The network-based methods can capture the implicit feature information in the topological structure of related biological heterogeneous networks containing lncRNAs, which is often ignored by sequence-based methods. In this paper, we summarize and discuss the materials, interaction score calculation algorithms, advantages and disadvantages of state-of-the-art algorithms of lncRNA–protein interaction prediction based on network methods to assist researchers in selecting a suitable method for acquiring more dependable results. All the related different network data are also collected and processed in convenience of users, and are available at https://github.com/HAN-Siyu/APINet/.

## 1. Introduction

Long non-coding RNAs (lncRNAs) are non-protein-coding transcripts with a length of more than 200 nucleotides, which can regulate gene expression at different levels [1]. LncRNAs were first regarded as transcriptional noise, and later it was found that they can play an important role in cell division, differentiation, metabolism and other physiological processes [2,3,4]. With the development of biotechnology and the emergence of computational models, there is now a great deal of evidence suggesting that lncRNAs are significant in diverse mechanisms and are involved in almost the whole process of cells from one division to the next [5,6], such as in transcriptional and post-transcriptional regulation, epigenetic regulation, tissue development, the process of genome selective expression in time and space and apotheosis, metabolic processes, cell cycle control and morphological and structural changes in chromosomes [7,8,9,10,11,12,13,14]. More and more reports have indicated that lncRNAs participate energetically in various stages of gene expression, including as signals, decoys, scaffolds, and leaders [15]. Compared with the characteristics of protein coding genes, lncRNAs tend to be less conserved across species and often show low expression level and high tissue specificity, which make the research more challenging and have attracted the attention of scientists and given rise to considerable discussions in recent decades.

Similar to protein-coding genes and microRNAs, lncRNAs have also been found in the regulation of many human complex diseases, including various types of cancer. At present, there are many databases of lncRNA associated with diseases, such as LncRNADisease database [16] and Lnc2Cancer database [17], which can be used to collect many kinds of disease-related lncRNA. The LncRNADisease database contains nearly 2000 lncRNA–disease associations, and Lnc2Cancer database contains 1488 lncRNA–cancer associations. It further confirms that lncRNA is closely related to diseases, even cancer and prognosis regulation. Obviously, the number of annotated lncRNAs involved in these two databases is relatively small compared with the number of identified lncRNAs, and most of the functions of lncRNAs associated with diseases are unclear. It is worth mentioning that lncRNA–protein interaction is a very important mechanism of lncRNAs. To fully understand function or molecular mechanism of lncRNAs, it is necessary to mine interactions between lncRNAs and other molecules, especially lncRNA–protein interactions.

It is of great importance to identify lncRNA–protein interactions to gain a comprehensive and profound understanding of the potential functions encompassed in their molecular mechanisms. At present, the main methods for identifying lncRNA–protein interactions are based on experimental approaches and computational approaches. Several large-scale experimental approaches for lncRNA- protein interaction prediction include RNAcompete [18], RNP immunoprecipitation-microarray (RIP-Chip) [19], high-throughput sequencing of RNA isolated by crosslinking immunoprecipitation (HITS-CLIP) [20] and photoactivatable ribonucleoside-enhanced crosslinking and immunoprecipitation (PAR-CLIP) [21]. There are also many effective methods for the analysis of experimental data, such as several methods for finding RNA motifs from crosslinking-immunprecipitation and high-throughput sequencing (CLIP-Seq) or other high-throughput experiments, such as BEAM (BEAr Motif finder) [22] and SMARIV (Sequence and Structure Motif enrichment Analysis for Ranked RNA daTa generated from In-Vivo binding experiments) [23]. NPInter database and StarBase database are built on these data, which are based on high-throughput experiments and have a certain degree of false positivity. True interactions may involve the integration of multi-source data, such as the STRING database containing PPI (protein–protein interactions), which integrates information from various sources, including experimental data, co-expression data, text mining, etc.

The methods of predicting lncRNA–protein interactions based on computational approaches are mainly divided into machine-based learning methods and network-based methods. The methods based on machine learning construct a classifier by fusing the features of sequence, structure and physicochemical properties, so as to form an interactive or non-interactive classification model. At present, the existing methods are RPISeq [24], de novo prediction [25], CatRAPID [26], LncPro [5], RPI-Pred [27] and rpiCOOL [28]. Random Forest, Nave Bayesian, Extended Nave Bayesian and SVM are the classifiers used in the above machine-based learning methods. There are also two methods to construct classification model based on deep learning: IPMiner [29] and lncADeep [30]. Current network-based approaches include LPBNI [31], fusing multiple protein–protein similarity networks (PPSNs) proposed by Zheng et al. [32], the method to predict lncRNA–protein interactions based on the relevance search method proposed by Yang et al. [33], LPIHN [34] and PLPIHS [35]. Compared to machine learning-based methods, network-based methods can accommodate more heterogeneous data, not only avoiding ignoring the external links between molecules, but also mining the hidden topological structure information in heterogeneous networks.

Nowadays, network science is being extensively used in biological and related fields. It provides many practical descriptions to characterize various biological systems [36] and the relationships between diseases and biological factors [37]. Network science is becoming more and more popular, and has achieved remarkable results in various fields of bioinformatics. Network science has also made rapid advances in disease gene prioritization [38], disease lncRNA prioritization [39,40,41], disease-related miRNA identification [42,43,44,45,46,47,48], disease metabolite prioritization [49] and drug–target interaction prediction [50,51,52]. In this paper, we focus on re-viewing network-based methods used for integrating heterogeneous data to predict lncRNA–protein interactions directly. The materials, interaction score calculation algorithms, and advantages and disadvantages of state-of-art algorithms of lncRNA–protein interaction prediction based on network methods are summarized and discussed to assist researchers in selecting a suitable method for acquiring more dependable results. This article is organized as follows. Section 2 summarizes the relevant databases used for analyzing lncRNA–protein interaction. Section 3 gives a brief introduction to experimental approaches and machine learning-based computational approaches for studying lncRNA–protein interaction. Section 4 systematically analyzes biological network-based computational models for lncRNA–protein interaction prediction. Section 5 includes the performance comparison of different network-based models for lncRNA-protein interaction prediction. And Section 6 briefly summarizes the discussion in this paper and looks forward to the future feasible methods.

## 2. A Brief Introduction to the Relevant Databases Used for Analyzing LncRNA–Protein Interactions

The various databases discussed in this article incorporate lncRNAs from different tissues and focus on lncRNAs as well as lncRNA-related interactions. Some of these databases are available at RNAcentral [53]. Although there is a great deal of overlapping sections among these databases, each database nonetheless offers considerable unique features. We present herein an overview of their respective contents and search features in order that researchers can get a quick glance of what each can offer. Then, we give a brief summary of the relevant databases mentioned in Table 1, including the name and website of the database and a brief description. We provide data information on all possible interactions between biomolecules that may be used in the research of lncRNA functions (which users can browse and download from https://github.com/HAN-Siyu/APINet/), that is, lncRNA–disease associations, lncRNA–lncRNA interactions, lncRNA–microRNA interactions, lncRNA–gene interactions, lncRNA–Gene Ontology (GO) interactions, microRNA–microRNA interactions, microRNA–disease associations, microRNA–gene interactions, microRNA–target interactions, gene–gene interactions, gene–metabolite interactions, metabolite–metabolite interactions, gene–GO interactions, gene–disease associations, gene–drug associations, metabolite–disease associations, drug–disease associations, drug–drug interactions, drug–side-effect interactions and and disease–disease interactions. The details of the data information are shown in Table 2. As some interaction data are integrated by multi-source data, in Table 2, we can see the types of these interactive data information, the number of sets of interaction data composed of several biological molecules and the sources of these data, which determine association data that can be used to construct heterogeneous networks, i.e., the composition of heterogeneous networks.

## 3. A Brief Introduction of Experimental Approaches and Computational Approaches Based on Machine Learning to Study LncRNA–Protein Interactions

### 3.1. LncRNA–Protein Interactions: From Experimental Approaches to Computational Models Based on High-Throughput Experiments

Several large-scale experimental approaches for lncRNA–protein interaction prediction include RNA immunoprecipitation (RIP) followed by mass spectrometry analysis, RNAcompete [18], RIP-Chip [19], high-throughput sequencing of RNA isolated by crosslinking immunoprecipitation (HITS-CLIP) [20], and photoactivatable ribonucleoside-enhanced crosslinking and immunoprecipitation (PAR-CLIP) [21]. Although these approaches can provide valuable data to construct a network of lncRNA–protein interactions, they are expensive and time-consuming, which are disadvantages that cannot be ignored. It is therefore urgent to put forward the computational approaches.

There are many effective methods for the analysis of experimental data, such as several methods for finding RNA motifs from CLIP-Seq or other high-throughput experiments, such as BEAM [22] and SMARIV [23]. BEAM is a method for structural motif discovery from a set of unaligned RNAs. Tested in various scenarios, BEAM is successful in retrieving structural motifs even in highly noisy datasets, such as those that can arise in CLIP-Seq or other high-throughput experiments. To solve the problem that the previous methods cannot provide information about protein structure preferences, the sequence and structure preferences of RNA-binding proteins can be inferred based on the feasibility of obtaining RNA structure information. SMARTIV is a novel computational tool for discovering combined sequence and structure binding motifs from in vivo RNA binding data relying on the sequences of the target sites, the ranking of their binding scores and their predicted secondary structures. The combined motifs are presented in a unified form, which is rich in information and easy for visual perception. These high-throughput experimental data can be used to predict the next step by developing machine learning methods. The quality of these models depends directly on the experimental data. At present, NPInter database and StarBase database are constructed from high throughput experimental data and are existing databases for lncRNA–protein interactions.

### 3.2. LncRNA–Protein Interactions: From Experimental Results to Computational Models Based on Machine Learning

Computational approaches for lncRNA–protein interaction prediction can be grouped into the following two ways of expressions. The first category is based on sequence and structural information and physicochemical properties, including RPISeq [24], de novo prediction [25], CatRAPID [26], LncPro [5], RPI-Pred [27], rpiCOOL [28], IPMiner [29] and lncADeep [30]. The second category is based on the fusion of heterogeneous data to construct a network, such as the lncRNA–protein bipartite network inference (LPBNI) method [31], fusing multiple protein–protein similarity networks (PPSNs) [32], the method to predict lncRNA–protein interactions based on the relevance search method proposed by Yang et al. [33], the prediction method of interactions between lncRNAs and proteins on heterogeneous networks (LPIHN) [34] and the predicting lncRNA–protein interactions using HeteSim scores (PLPIHS) method [35].

From the point of view of characteristics such as sequence information, various classical methods have been proposed. RPISeq [24] is proposed to predict RNA–protein interactions only using sequence information. The support vector machine (SVM) classifier and the random forest (RF) classifier, which are supervised machine learning algorithms, are used in the RPISeq. De novo prediction of RNA–protein interactions [25] also only considers sequence information. A set of known RNA–protein interactions is collected as gold-standard positives, where sequence-based features are extracted for each RNA–protein pair [25]. In the process of constructing the Bayes classifier, these effective features are used to train an RNA–protein interaction prediction model. CatRAPID [26] is proposed by using physicochemical properties, including the secondary structures of the molecules and their propensities for hydrogen bonding and van der Waals interactions. Encoding the protein–RNA pairs into feature vectors is the first step, followed by calculating the interaction score through the matrix computation. LncPro [5] is proposed to predict ncRNA–protein interactions by using Fisher’s linear discriminant approach. The training features are not only from protein secondary structures and their propensities for hydrogen bonding and van der Waals interactions, but also from RNA secondary structures [93]. LncPro also requires the identification of a matrix and calculation of the interaction score to represent degree of interactions through matrix computation by a simple machine-learning model for matrix computation. RPI-Pred [27], a SVM-based machine-learning approach, is proposed by considering sequence features and combining the high-order structures of both proteins and RNAs. This interaction prediction considers protein blocks rather than classical three-state protein secondary structures. Five classes of RNA secondary structures are regarded as high-order structures. RpiCOOL [28] is a tool developed for detecting RNA–protein interactions in silico by using the RF classifier, which classifies RNA and protein based on whether there are interactions between them. The sequence composition and repetitive patterns are used as heterogeneous information of the protein and RNA, which is then used to encode feature vectors to express pairs between RNA and protein. IPMiner [29], a tool based on simple sequence composition features, integrates deep neural network and stacked ensembling classifiers to identify RNA–protein interactions. The extracted original features, SDA (stacked denoising autoencoder) and SDA-FT (SDA with fine tuning), are provided to the RF classifier, respectively. The outputs of these three classifiers, which are trained by a logistic regression mode, are integrated through superposition. These computational methods fill the broadening gap between raw and annotated data that has been generated as a result of the large amount of data obtained by high-throughput technologies. LncADeep is proposed to predict lncRNA-protein interactions based on deep neural networks, using both sequence and structure information.

With the development of computational approaches, experimental methods are now suffering the great disadvantage to predict lncRNA–protein interactions, such as high cost and long time. Intrinsic features of lncRNA and protein have increasingly interested the researchers. The advantage of intrinsic features has been demonstrated in the research of lncRNA identification. The methods of lncRNA–protein interaction prediction focus on intrinsic features of lncRNA and protein, such as sequence information, structure information, and physicochemical properties, including hydrogen-bond and van der Waals propensities. We analyzed the dataset of methods based on what kind of information they use, such as sequence, structure and physicochemical information. We also analyzed what machine learning algorithms are employed in the different methods. The comparison of each method is shown in Table 3. In this article, we give a more detailed introduction to each computational model for lncRNA–protein interaction prediction based on intrinsic features of lncRNA and protein. To make it easier for users to use these computation models, we have supplemented the availability network resources. We give more details about each computational method’s availability, such as the web server or offline package for lncRNA–protein interaction prediction based on sequence and structural information and physicochemical properties.

Whereas the machine learning-based methods only consider the properties of the RNAs or proteins and neglect interactions between lncRNAs and proteins, the network-based methods pay more attention to this kind of interactions, which are implicated in the topologies between nodes in the heterogeneous networks of lncRNAs. When the sequence is too long or the randomness of structural information is predicted, the computational models based on machine learning will be affected to some extent.

## 4. Computational Models for LncRNA–Protein Interaction Prediction Based on Biological Networks

**Figure 1:**
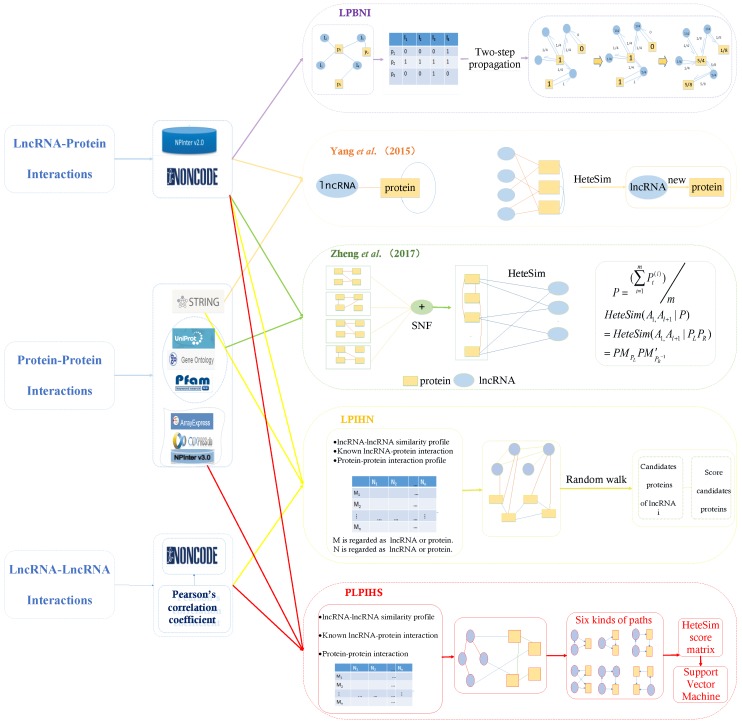
Overview of five computational models for lncRNA–protein interaction prediction based on network method, including data collection and core algorithm. Illustration: The specific algorithm implementation of each method is represented by rectangular boxes with dotted lines of different colors, and the solid lines with different colors outside the rectangular boxes of dotted lines represent the data sources used by different methods. These colors are the same as the colors used by method names. In addition, the solid line color in the dotted rectangular frame is used to distinguish the interaction of lncRNA–lncRNA, protein–protein or lncRNA–protein.

The previously described methods for predicting the interactions between lncRNAs and proteins more focus on the intrinsic features of lncRNAs and proteins but do not take the topological structures of biological networks associated with the lncRNAs into consideration. A biological network can apply to biological systems. Nowadays, network science is being used extensively in the biological and related fields. Network science provides many practical descriptions of biological systems and relationships between diseases and other biomolecules as biological factors [33]. Moreover, we could integrate known lncRNA–protein interaction networks, lncRNA–lncRNA similarity networks and PPI networks that were downloaded in the databases and fused by multiple PPSNs to construct heterogeneous networks and implement a model based on computing node similarity between networks to discover possible interactions between lncRNAs and proteins, such as random walk on heterogeneous networks and kinds of propagation algorithms that can discover potential associations. The overview is presented in Figure 1. We analyzed which heterogeneous data are selected by each method, how to fuse heterogeneous data to construct the network, and what methods are used to deal with heterogeneous networks to predict lncRNA–protein interactions. We analyzed the differences among the different network-based methods such as the datasets that are used in each method, how to fuse heterogeneous data to construct the network and algorithms for specific computation interactions. The differences of each network-based method are shown in Table 4. In this articl, we give a more detailed introduction to each computational model for lncRNA–protein interaction prediction based on biological networks.

### 4.1. LPBNI: A Bipartite Network-Based Method for the Prediction of LncRNA–Protein Interactions

**Figure 2:**
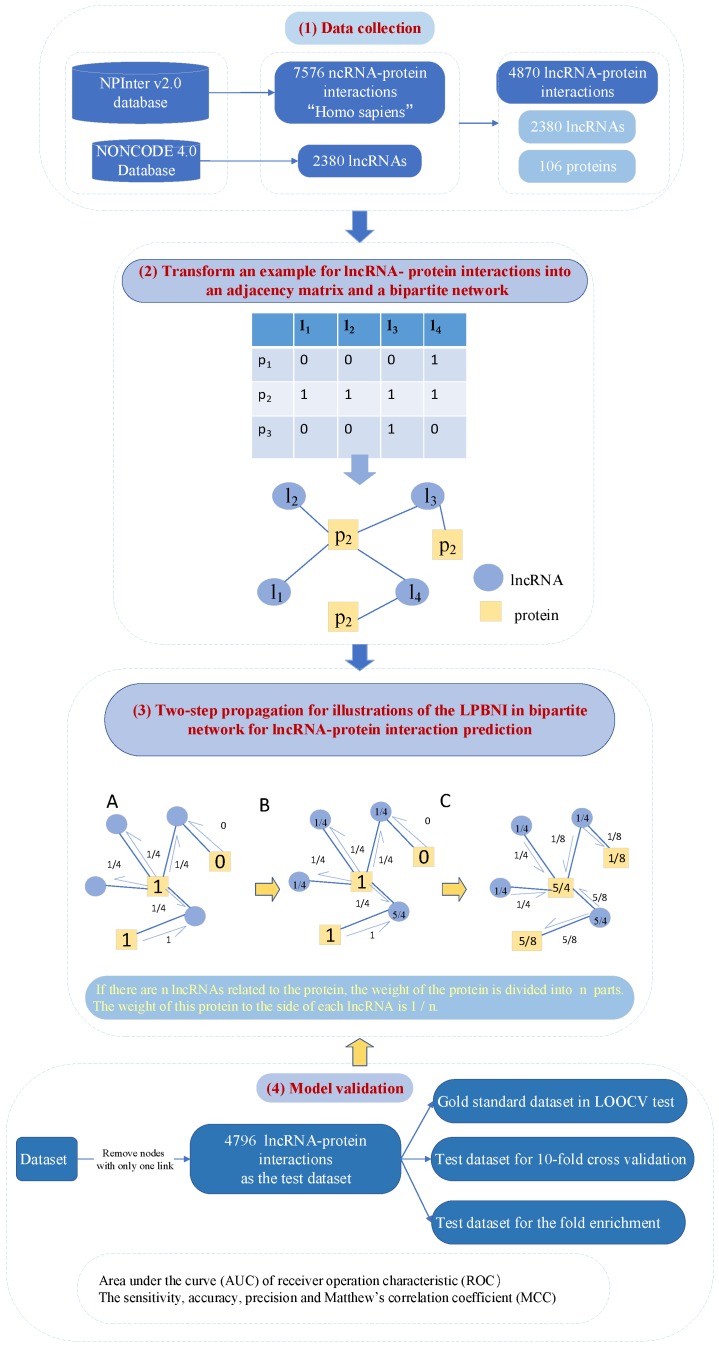
Framework of LPBNI mainly including four modules: (**1**) Data collection: the lncRNA–protein interaction network is from NPInter and NONCODE. (**2**) Bipartite network construction (a toy example in Figure 1). (**3**) Two-step propagation on the bipartite network: (**A**) The process of the initial information propagated from proteins to their direct neighbor lncRNAs. For example, the initial information of three proteins is 1, 1 and 0, respectively. (**B**) The score on red circles is the information of each lncRNA received from proteins. (**C**) The process of the information propagated from lncRNAs back to proteins. The score on blue hexagon in (**C**) is the final information of each protein after the two-step propagation. The red circles represent lncRNAs and the blue hexagons represent proteins. (**4**) Model validation based on leave one out cross validation (LOOCV), the area under the receiver operating characteristic curve (AUC) and Matthew’s correlation coefficient (MCC).

Inspired by resource methods in dynamically allocated networks, Zhou et al. [96] proposed algorithms based on the propagation process of the LPBNI method. Li et al. [34] developed this method on the basis of an lncRNA–protein bipartite network to predict lncRNA–protein interactions. A graph *G* can be used to represent the lncRNA–protein interaction network. The structure of the bipartite network of lncRNA–protein is simply shown in graphic language, as shown in Figure 2. Finally, they chose to apply the propagation method on the constructed network and calculated the degree of lncRNA–protein interactions as a score. In the G(L,P,E), the propagation matrix is used as *W*, where $W_{ik}$ represents the information transferred from the $p_{k}$ node to the $p_{i}$ node, and the transmission of key information between two nodes represents the importance of nodes. For each lncRNA $l_{j}$, they defined $S_{0}\left(i\right)=s_{ij},i\in \left\{1,2,\ldots ,m\right\}$ as the first information on protein *P*, where $s_{ij}=1$ if $p_{i}$ interacts with $l_{j}$; otherwise, $s_{i,j}=0$. $S_{L}\left(l_{j}\right),j\in \left\{1,2,\ldots ,n\right\}$ represents the score on $l_{j}$ after the first step of information propagation, which can be calculated as

(1) $$S_{L}=\sum_{i=1}^{m}\frac{a_{ij}S_{0}\left(i\right)}{d(p_{i})}.$$

In the formula above, $d\left(p_{i}\right)=\sum_{j=0}^{n}a_{ij}$ is the number of lncRNAs that interact with $p_{i}$.

In the next step, all the information in *L* propagates back to *P*. $S_{F}\left(p_{i}\right)$ is defined as the final information on protein $p_{i}$, signifying the interaction score of protein $p_{i}$ with $l_{j}$. $S_{F}$ can be defined as

(2) $$S_{F}\left(i\right)=\sum_{j=1}^{n}\frac{a_{ij}S_{L}\left(l_{j}\right)}{d(l_{j})}=\sum_{j=1}^{n}\frac{a_{ij}}{d(l_{j})}\sum_{k=1}^{m}\frac{a_{kj}S_{0}\left(k\right)}{d(p_{k})},$$

where $d\left(l_{j}\right)=\sum_{l=0}^{m}a_{ij}$ is the number of proteins that interact with $l_{j}$. The final information $S_{F}$ can be defined in the matrix as

(3) $${\vec{S}}_{F}=W{\vec{S}}_{0},$$

where ${\vec{S}}_{0}$ is the column vector of $S_{0}$, and ${\vec{S}}_{F}$ is the final score of the lncRNA that users query after the two-step information propagation in the lncRNA–protein interaction network. $S_{F}$ can be represented as

(4) $$S_{F}\left(i\right)=\sum_{k=1}^{m}w_{ik}S_{0}\left(k\right),W_{ij}=\frac{1}{d(p_{i})}\sum_{j=1}^{n}\frac{a_{ij}a_{kj}}{d(l_{j})}.$$

After calculations, the protein sorted by the final score $S_{F}$ for $l_{j}$ is obtained. All the candidate proteins are ranked in decreasing order, and proteins with a high ranking are considered to interact with lncRNA $l_{j}$.

LOOCV was performed on the heterogenous network containing lncRNA–protein interactions, leaving only one sample for the test set at a time, and the other samples were used as the training set. Although the calculation was more complicated than other verification methods, the sample utilization rate was the highest. LOOCV aws used to evaluate the performance of the proposed method. In the course of the calculation, each lncRNA–protein pair was omitted as a test sample by changing the value in the adjacency matrix *A* to 0. The performance of LPBNI could be estimated by the ratio of its predicted interactions to the originally known lncRNA-protein. A receiver operating characteristic (ROC) curve was selected as a criterion to evaluate the LPBNI and random walk with restart methods. The propagation matrix *W* proposed in the LPBNI method is dependent on the adjacency matrix *A* of the bipartite network. When applying LOOCV, multiple values of W were obtained, owing to the change of *A* values during each step of the cross-validation. Consequently, the value of *W* was recalculated for each lncRNA–protein pair that was left out as a test sample. In addition, nodes that do not propagate information are not considered when evaluating the performance of the method, where nodes with fewer than two links are defined as nodes that do not propagate information in the process of cross-validation.

### 4.2. Fusing Multiple Protein–Protein Similarity Networks to Effectively Predict LncRNA–Protein Interactions

**Figure 3:**
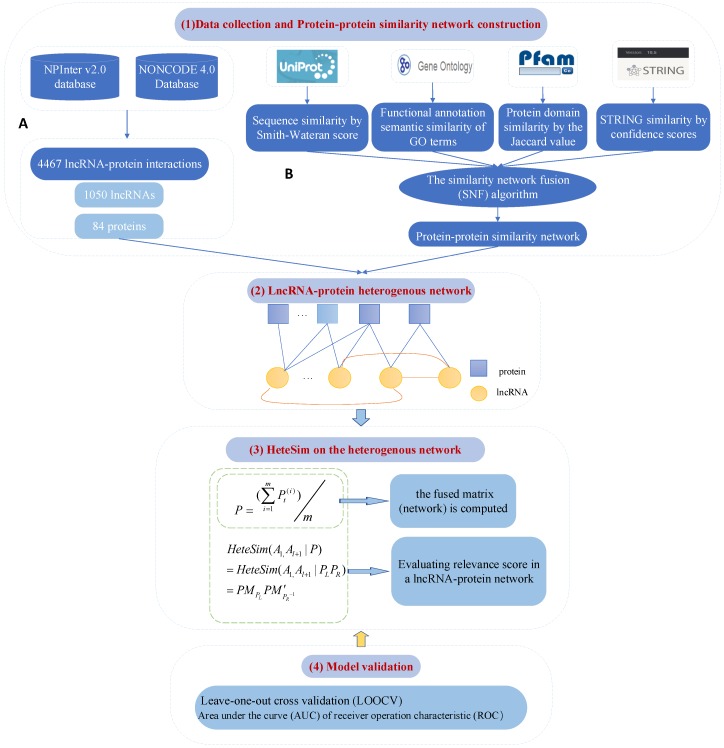
Framework of the proposed method by Zheng et al. [32] mainly containing four modules. (**1**) (**A**) Data collection: The lncRNA–protein network is from NPInter and NONCODE. The datasets from Uniprot, GO, Pfam and STRING database are collected for protein–protein similarity network construction. (**B**) Protein–protein similarity network construction: based on similarity network fusion (SNF) algorithm by integration of multi-resource information. (**2**) A heterogeneous network construction. (**3**) HeteSim computation on the heterogeneous network. (**4**) Model validation based on LOOCV and AUC.

To improve the performance of lncRNA–protein interaction prediction, Zheng et al. [32] fused multiple PPSNs to construct a multilevel heterogeneous network. New lncRNA–protein interaction predictions are inferred by integrating the fused PPSNs with known lncRNA–protein interaction predictions (Figure 3). Protein sequences, protein domains, GO terms, and the STRING database are first used to construct four Protein–Protein Similarity Networks (PPSNs), following which the SNF algorithm [95] is employed to combine the four protein–protein similarity networks into a fused protein–protein similarity network. Then, a heterogeneous lncRNA–protein network is built including based on the fused protein–protein similarity network and the known lncRNA–protein interactions. Finally, the HeteSim algorithm [94] is used to infer new lncRNA–protein interaction predictions. Extensive experiments show that this approach outperforms not only the existing methods for predicting the lncRNA–protein interactions, but also performs well by using only one PPSN as a protein–protein interaction network without fusing four different aspects of the protein–protein similarity network into a protein–protein interaction network. After fusing all the four matrices, the area under the curve (AUC) value of 0.9068 indicates the best performance. This result shows that a more reliable and informative network can be obtained by fusing multiple matrices.

The advantage of SNF algorithm is that it can obtain valuable information from a relatively small number of samples, and it has strong robustness in dealing with noise and data heterogeneity. It is a nonlinear method based on the typical nature of the complexity of the natural world based on message-passing. The nonlinear method is closer to the nature of the objective thing itself. It is one of the important methods to quantitatively study and understand complex knowledge. This method iteratively updates each network and makes it more and more similar to other networks. A protein similarity network can be represented as a graph G=(V,E), where $V=\{v_{1},v_{2},\ldots ,v_{n}\}$ represents a set of corresponding proteins in the network, and *E* represents a set of edges, each of which has a similarity weight. The authors denoted the corresponding similarity matrix as *W*, where W(i,j) is the similarity between proteins $v_{i}$ and $v_{j}$. They defined a full and sparse kernel on each matrix in order to compute the fused network from four protein similarity matrices. The full kernel is a normalized weight matrix $P=D^{-1}W$, where *D* is a diagonal matrix and $D\left(i,j\right)=\sum_{j}W\left(i,j\right)$. Because *P* involves self-similarities on the diagonal entries of *W*, a better form for avoiding numerical instability is as follows [96]:

(5) $$P\left(i,j\right)=\left\{\begin{array}{cc} \frac{W(i,j)}{2\sum_{k\neq i}W\left(i,k\right)}, & j\neq i\\ 1/2, & j=i. \end{array}\right.$$

Protein $v_{i}$’s neighbors are denoted as $N_{i}$ and use *k* nearest neighbors (*k*NN) to measure the local part as follows:

(6) $$S\left(i,j\right)=\left\{\begin{array}{cc} \frac{W(i,j)}{\sum_{k\in N_{i}}W\left(i,k\right)}, & j\in N_{i}\\ 0, & \mathrm{otherwise}. \end{array}\right.$$

A protein has much better similarities to its neighbors than it has to remote proteins. Similarity based on graph diffusion principle can be propagated to remote proteins. Matrix *P* provides all the information of the PPSN, whereas *S* provides the local similarity information of the network. Then, iterative computation can occur as follows:

(7) $$P_{t}^{\left(i\right)}=S^{\left(i\right)}\times \left(\frac{\sum_{k\neq i}P_{t-1}^{\left(k\right)}}{m-1}\right)\times {\left(S^{\left(i\right)}\right)}^{T},i=1,2,3,4,$$

where $P_{t}^{\left(i\right)}$ is the *i*th similarity matrix after t(≥0) iterations, and $S^{\left(i\right)}$ is the *k*NN matrix of the similarity matrix or network. Following that, *m* is the number of PPSNs used. As *S* is the *k*NN matrix of *P*, it contains the most important information of *P* and also alleviates the noise effect of *P*. In each iteration, each similarity matrix can get more reliable information from other similarity matrices, at the same time, it will update its own matrix based on other similarity matrices. After *t* iterations, the fusion network can be replaced by a fusion matrix, which is defined as follows:

(8) $$P=\left(\sum_{i=1}^{m}P_{t}^{\left(i\right)}\right)/m.$$

Note that the authors normalized matrix $P_{t}$ after each iteration, each protein has a higher degree of similarity to itself in order to ensure that the matrix is in a full rank state than other proteins. With the known lncRNA–protein interactions and the fused PPSN, they built a lncRNA–protein heterogeneous network, on which a random walk model HeteSim was used to infer new lncRNA–protein interactions. HeteSim is used to evaluate the relevance between a lncRNA–protein pair, where a large relevance score means the lncRNA and protein have more interactions.

For this method, 15 settings made up of different combinations of the similarity matrices (Seqs, Pfam, GO, and STRING, respectively) were implemented. The path selection is very important since HeteSim is a path-constrained relevance measure. In the fusion work, the relevance path was chose as lncRNA-protein-protein, which was the same as that used in the work of Yang et al. [33]. With the proof of the experiment and more matrix merging, the AUC value becomes more ideal. For example, the AUC value of GO + Pfam + STRING is 0.9066, which is larger than the AUC value of GO + Pfam, GO + STRING and Pfam + STRING. When all four protein similarity matrices were fused, AUC achieved the best result of 0.9068. This shows that, with the increase of the number of fusion matrices, we could get more specific information of protein similar network. This multi-matrix fusion method is convenient to get more reliable and informative data representation.

### 4.3. Prediction of Interactions between lncRNA and Protein by Using Relevance Search in a Heterogeneous LncRNA–Protein Network

**Figure 4:**
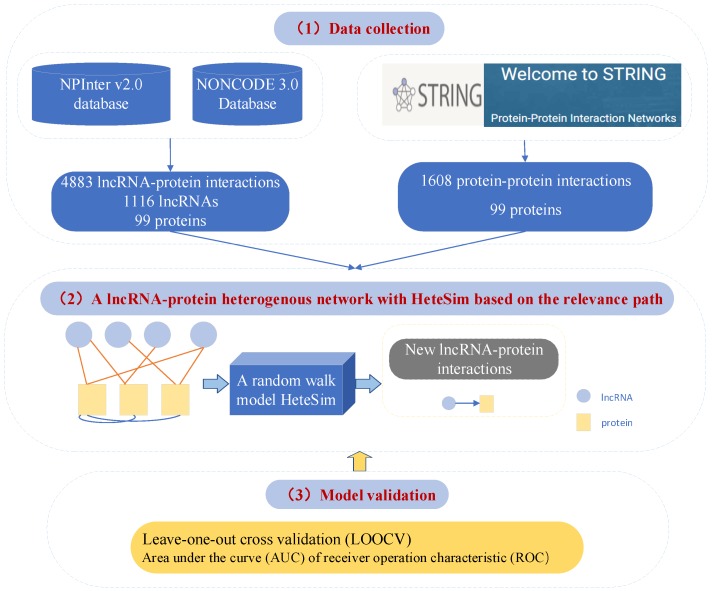
Pipeline of the method proposed by Yang et al. [33]. (**1**) Data collection: lncRNA–protein interactions from NPInter and NONCODE and protein–protein interactions from STRING database. (**2**) HeteSim computation based on relevance path of heterogenous network for lncRNA–protein interaction predictions. (**3**) Model validation based on LOOCV and AUC.

Yang et al. [33] tried to use the possible hidden information in the biological network topologies containing lncRNA layer networks. Thus, an algorithm named HeteSim is introduced to measure the relevance between lncRNAs and proteins on the basis of the heterogeneous lncRNA–protein network, which integrates the known lncRNA–protein interaction networks and PPSNs. Figure 4 shows a network model and the schema of the interaction network. The AUC of HeteSim for the lncRNA MALAT1 is 0.955. The performance results of network-assisted method confirm a difficult problem. It is difficult to break through the low conservatism of lncRNAs by traditional methods to predict the interactions between lncRNAs and proteins, which is a challenge to propose new methods to predict lncRNA–protein interactions, which generally uses information from intrinsic features of the RNA and protein alone. Their approach also demonstrates the tremendous value of the network-based approach in lncRNA-related fields, and has valuable implications for predicting interactions in heterogeneous networks constructed from biomolecules.

In the HeteSim algorithm [94], relevance paths are defined. A relevance path *P*, denoted as $A_{1}\overset{R_{1}}{⟶}\ldots \overset{R_{l}}{⟶}A_{l+1}$, is a path defined over the schema $T_{G}=\left(A,R\right)$. A composite relation $R=R_{1}\circ R_{2}\circ \ldots \circ R_{l}$ between node types $A_{1}$ and $A_{l+1}$ is revealed by the symbolization of the relevance path, where ∘ denotes the composition operator of relations. For a given relevance path $R=R_{1}\circ R_{2}\circ \ldots \circ R_{l}$, HeteSim can measure the similarity between two objects *x* and *y* ($x\in R_{1}.X\;\mathrm{and}\;y\in R_{1}.Y$) according to the relevance score:

(9) $$\begin{array}{c} HeteSim\left(x,y|R_{1}\circ R_{2}\circ \ldots \circ R_{l-1}\circ R_{l}\right)=\frac{1}{\left|O\left(x|R_{1}\right)||I\left(y|R_{l}\right)\right|},\\ \sum_{I(v|R_{l})}^{O(x|R_{1})}HeteSim\left(O_{i}\left(x|R_{1}\right),I_{j}\left(y|R_{l}\right)|R_{1}\circ R_{2}\circ \ldots \circ R_{l-1}\circ R_{l}\right). \end{array}$$

$O(x|R_{1})$ represents the out-neighbors of *x* based on relation $R_{1}⋃R_{2}$, and $I(y|R_{l})$ represents the neighbors of *y* based on relation $R_{l-1}\circ R_{l}$. In fact, *x* and *y* can also be the same type according to the random walk model pair. For an arbitrary relevance path $P=A_{1}A_{2}\ldots A_{l+1}$, the HeteSim relevance between any two objects $a\in A_{1}$ and $b\in A_{l+1}$ is the corresponding component in the score matrix named HeteSim $\left(A_{1},A_{l+1}|P\right)$. Finally, the relatedness between $A_{1}$ and $A_{l+1}$ in the relevance path $P=A_{1}A_{2}\ldots A_{l+1}$ is defined as follows:

(10) $$\begin{array}{c} HeteSim(A_{1},A_{l+1}|P)\\ =HeteSim(A_{1},A_{l+1}|P_{L}P_{R})\\ =PM_{P_{R}}*PM_{P_{R}^{-1}}^{\prime }\\ =U_{A_{1}A_{2}}\cdots U_{A_{mid-1}M}V_{MA_{mid+1}}\cdots V_{A_{l}A_{l+1}}\\ =U_{A_{1}A_{2}}\cdots U_{A_{mid-1}M}U_{A_{mid+1}M}^{\prime }\cdots U_{A_{l+1}A_{l}}^{\prime }\\ =U_{A_{1}A_{2}}\cdots U_{A_{mid-1}M}\left(U_{A_{l+1}A_{l}}\cdots U_{A_{mid+1}M}\right). \end{array}$$

Based on the random walk model [37], *P* is divided into two equal path lengths $P_{L}$ and $P_{R}$, where $P_{L}=A_{1}A_{2}\cdots A_{mid-1}M$ and $P_{R}=MA_{mid+1}\cdots A_{l+1}$. Depending on whether the length of *P* is even or odd, the node type of *M* is impacted differently. If the length of *P* is even, *M* is the middle position node type, which could be one of *A*. Otherwise, it is just the defined middle type. $P_{R}$ is equal to $P_{L}^{-1}$. The transition probability matrix of $A_{i}\to A_{j}$ denoted as $U_{A_{i}A_{j}}$ is the normalized matrix of the adjacent matrix $W_{A_{i}A_{j}}$ that contains the row vector, and the transition probability matrix of $A_{i}\to A_{j}$ denoted as $V_{A{}_{i}A{}_{j}}$ is the normalized matrix of $W_{A_{i}A_{j}}$ that contains the column vector. It easily proves that $V_{A{}_{i}A{}_{j}}$ is equal to $U_{A{}_{i}A{}_{j}}^{\prime }$. Finally, the score between two objects is normalized to ensure that the correlation between the same objects is 1. Based on HeteSim algorithm in the heterogeneous network of lncRNA–protein, the lncRNA–protein-related pathway is considered. In this network, a group of data is randomly extracted from the measured data as a training dataset, and the rest of the data are used as the test dataset. The AUC of HeteSim achieved on the lncRNA–protein–protein path is 0.879.

### 4.4. LPIHN: LncRNA–Protein Interaction Prediction Based on Heterogeneous Network Models

**Figure 5:**
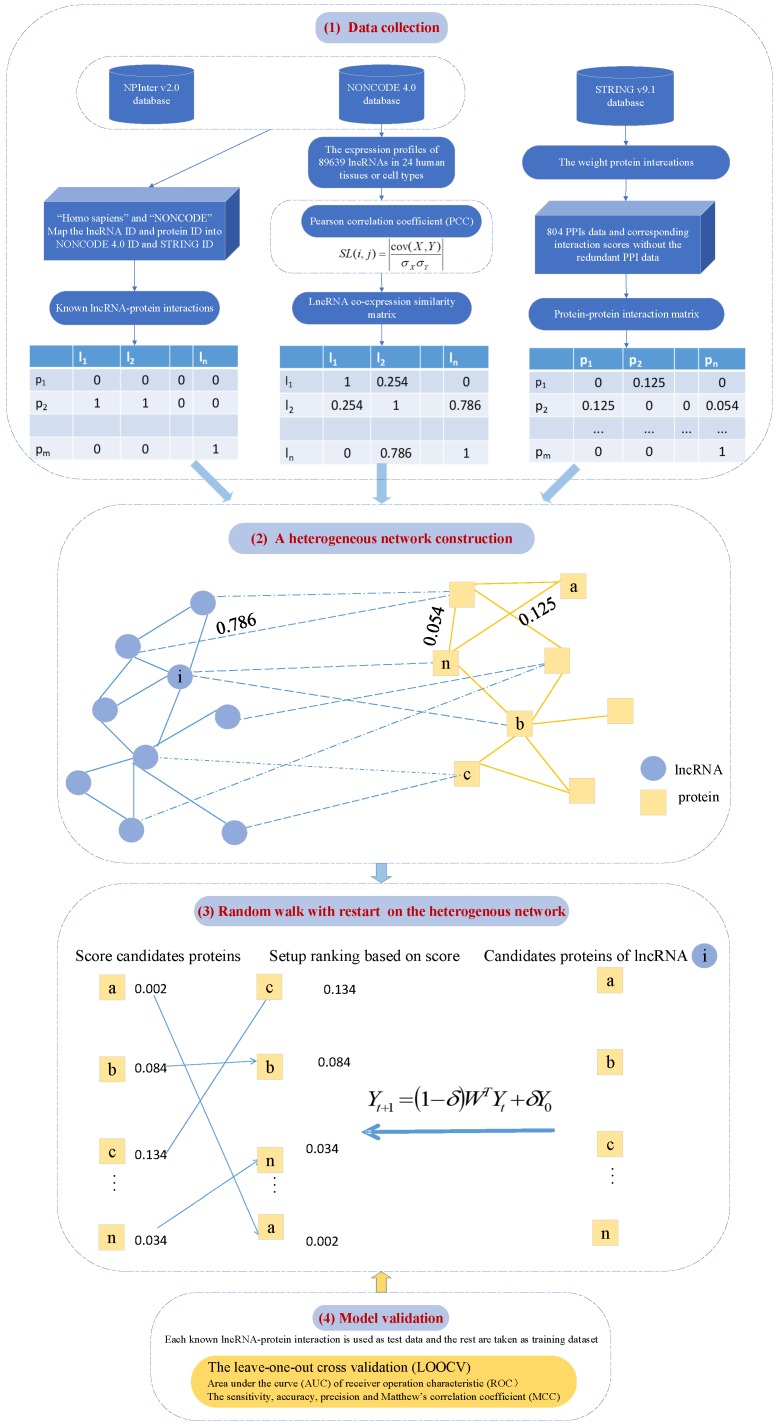
Pipeline of LPIHN, containing three modules: (**1**) Data collection: lncRNA–protein interactions from NPInter, protein–protein interactions from STRING database and lncRNA–lncRNA similarity network computed based on lncRNA expression profile from NONCODE. (**2**) A heterogeneous network construction. (**3**) LncRNA–protein interactions prediction based on the random walk with restart. A score is assigned to each candidate protein of a query lncRNA, by the random walk with restart on the heterogeneous network. The candidate proteins are ranked based on the scores. (**4**) Model validations based on LOOCV and AUC. For LPIHN, the lncRNA–lncRNA similarity network is calculated by using the lncRNA expression profiles based on the PCC of each pair of lncRNAs. The heterogeneous network is constructed by connecting the lncRNA–lncRNA similarity network and PPI network together with the known lncRNA–protein interaction network. Blue circles indicated lncRNAs, orange squares indicated proteins, blue edges indicated lncRNA–lncRNA similarities, orange edges indicated protein–protein interactions, and blue dotted edges indicated known lncRNA–protein interactions.

Based on this assumption, interrelated lncRNAs tend to exhibit interaction patterns that have similarities with proteins. Li et al. [34] proposed the network-based computational method LPIHN for predicting new lncRNA–protein interactions. The LPIHN procedure is shown in Figure 5. A heterogeneous network is constructed, which is integrated by a similarity network containing lncRNA–lncRNA expression data, a lncRNA–protein interaction network and a PPI network. The similarity network containing lncRNA–lncRNA expression data is calculated by the Pearson’s correlation coefficient [97,98,99,100,101,102] between the expression profiles of each lncRNA–lncRNA interaction. The lncRNA–protein interaction network is constructed from NPInter, by Shang et al. [103], who made a detailed and comprehensive analysis of it. The protein–protein interaction network is not a single source; it is based on computational prediction methods, and some of the interaction data are obtained through high-throughput experiments, from the STRING v9.1 database [104] to text mining, data obtained from the three weighted protein interaction degrees. Then, they walk randomly over the heterogeneous network to infer and predict the interaction between new lncRNAs and proteins.

In the RWR procedure [37], an iterative walker starts at a source node with the first probability, and then it can either move to a randomly selected direct neighbor in the process of random walking or restart at a source node with probability δ in each step. Therefore, when random walks are completed on heterogeneous networks, researchers can determine the initial probability, transfer matrix, and restart probability. However, it is based on information provided by heterogeneous networks. During the process of predicting the potential proteins for lncRNA $l_{i}$, $Y_{0}$ represents the first probability of the walker starting at every node, where $l_{i}$ and the proteins that are known to interact with $l_{i}$ are assigned positive values, and the nodes that remain are assigned as zero. It means that the node where the random walk begins is $l_{i}$, or that the protein interacts with $l_{i}$. $Y_{i}$ represents the relevance of $l_{i}$ to all other nodes, where *j* represents the node and *t* represents the step. $Y_{t+1}$ can be defined by the following equation:

$$Y_{t+1}=\left(1-\delta \right)W^{T}Y_{t}+\delta Y_{0},$$

where δ∈(0,1) represents the restart probability of the random walk. *W* is the transition matrix and $Y_{0}$ is the first probability of the random walk. For a given lncRNA $l_{i}$, $l_{i}$ is the seed node in the lncRNA network, the probability of vertex $l_{i}$ is 1, and other elements in the lncRNA network are assigned as zero, which forms the first probability of the lncRNA network $v_{0}$. When protein $p_{j}$ interacts with lncRNA $l_{i}$, $p_{j}$ becomes the seed node in the protein network. The first probability vector of the protein network $u_{0}$ is formed by assigning equal probabilities to the protein seed nodes. For the heterogeneous network, the first probability is

(11) $$Y_{0}=\left[\begin{array}{c} \left(1-\beta \right)u_{0}\\ \beta v_{0} \end{array}\right].$$

The parameter β∈(0,1) can decide whether to focus more on lncRNA networks or more on protein networks. When β=0.5, failure to focus more on one side of a similar network means that the lncRNA–lncRNA similarity network and the PPI network are given the same weight. With β<0.5, the random walk tended to return to the protein network. The transition matrix was defined in order to complete the random walk on the heterogeneous network. The authors defined $W=\left[\begin{array}{cc} W_{P} & W_{PL}\\ W_{LP} & W_{L} \end{array}\right]$ as the transition matrix, where $W_{P}$ is the subnetwork transition matrix showing the probability of the random walker transiting between the protein and another protein in the random walking process. $W_{L}$ between lncRNA and another lncRNA can be calculated in a similar way. $W_{PL}$ represents the probability of the random walker transiting from the protein network to the lncRNA network, and $W_{LP}$ represents the relationship of the lncRNA network to the protein network. In the process of transition, they defined γ as the probability of the random walker transiting from the protein network to the lncRNA network, where the reverse is also true. *W*, the probability of the random walker transiting from protein $p_{i}$ to $p_{j}$, is defined as

(12) $$W_{P}\left(i,j\right)=p\left(p_{j}|p_{i}\right)=\left\{\begin{array}{cc} \frac{SP^{\prime }\left(i,j\right)}{\sum_{j}SP^{\prime }\left(i,j\right)}, & \sum_{k}I\left(i,k\right)=0\\ \frac{\left(1-\gamma \right)SP^{\prime }\left(i,j\right)}{\sum_{j}SP^{\prime }\left(i,j\right)}, & \mathrm{otherwise}, \end{array}\right.$$

where $\sum_{k}I\left(i,k\right)=0$ means that $p_{i}$ can bind to multiple lncRNAs and at least one lncRNA, and can be transferred from $p_{i}$ to a similar network of lncRNA–lncRNA at random. In this case, the probability with γ of $p_{i}$ transferring to $l_{i}$ can be further calculated. The probability of $p_{i}$ transiting to $p_{j}$ should multiply 1−γ. The probability of transiting from lncRNA $l_{i}$ to $l_{j}$ can be defined as:

(13) $$W_{L}\left(i,j\right)=p\left(l_{j}|l_{i}\right)=\left\{\begin{array}{cc} \frac{SL(i,j)}{\sum_{j}SL\left(i,j\right)}, & \sum_{k}I\left(k,i\right)=0\\ \frac{\left(1-\gamma )SL(i,j\right)}{\sum_{j}SL\left(i,j\right)}, & \mathrm{otherwise}. \end{array}\right.$$

The probability of transiting from protein $p_{i}$ to lncRNA $l_{j}$ is defined as

(14) $$W_{PL}\left(i,j\right)=p\left(l_{j}|p_{i}\right)=\left\{\begin{array}{cc} \frac{\gamma I(i,j)}{\sum_{j}I\left(i,j\right)}, & \sum_{k}I\left(i,k\right)\neq 0\\ 0, & \mathrm{otherwise}, \end{array}\right.$$

where $\sum_{k}I\left(i,k\right)\neq 0$ means that $p_{i}$ is bound to at least one lncRNA, and the walker can transit to the lncRNA–lncRNA network from $p_{i}$ with probability γ; under that condition, one can further calculate the probability of $p_{i}$ transiting to $l_{j}$. The probability of transiting from lncRNA $l_{i}$ to protein $p_{j}$ can be defined in a similar manner as

(15) $$W_{LP}\left(i,j\right)=p\left(p_{j}|l_{i}\right)=\left\{\begin{array}{cc} \frac{\gamma I(j,i)}{\sum_{j}I\left(i,j\right)}, & \sum_{k}I\left(k,i\right)\neq 0\\ 0, & \mathrm{otherwise}. \end{array}\right.$$

As the first probability $Y_{0}$ and the transition matrix *W* were defined, the RWR procedure [37] could be used for the heterogeneous network. After multiple iterations, the change between $Y_{t}$ and $Y_{t+1}$ was less than $10^{-10}$, which meant that a stable probability $Y_{\infty }={\left[\left(1-\beta \right)u_{\infty },\;\beta v_{\infty }\right]}^{T}$ had been obtained.

The result of the LOOCV test showed that the approach could achieve 0.96 with an AUC value. Some predicted interactions between lncRNAs and proteins have been confirmed in recent research studies and databases, indicating the strong influence of LPIHN in predicting lncRNA–protein interactions. In each cross-validation experiment, the test dataset was associated with each known lncRNA–protein interaction, while the rest was used as a training dataset. The method has been successfully reconstructed and possible interactions have been evaluated. In particular, the authors use curves and fold enrichment to measure performance, and it is worth mentioning that the average-fold enrichment of all test data is also used to evaluate the model.

### 4.5. PLPIHS: Prediction of LncRNA–Protein Interactions Using HeteSim Scores Based on Heterogeneous Networks

**Figure 6:**
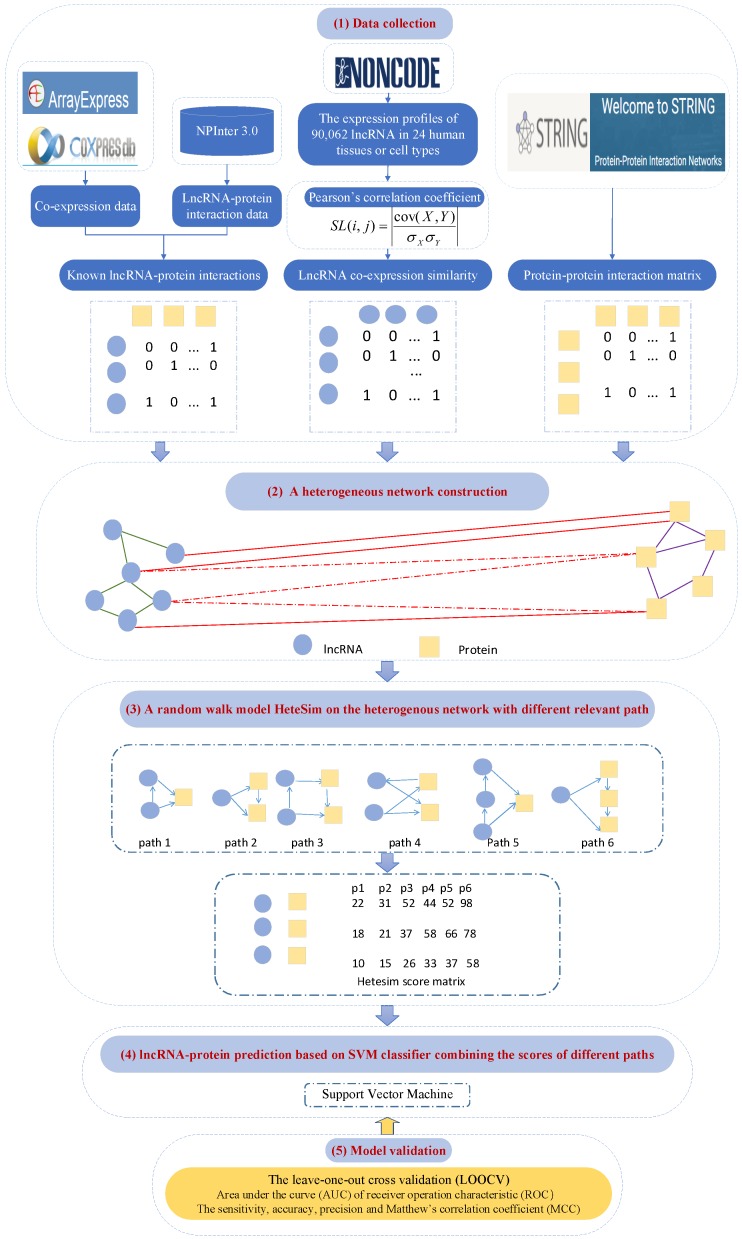
Flowchart of PLPIHS, including four modules: (**1**) Data collection. (**2**) Heterogeneous network construction consisting of a lncRNA–lncRNA similarity network, a lncRNA–protein interaction network and a protein–protein interaction network. (**3**) HeteSim measure is used to calculate a score for each lncRNA–protein pair in each path. (**4**) LncRNA–protein prediction based on SVM classifier combining the scores of different paths. (**5**) Model validations based on LOOCV, AUC and MCC.

Predicting the association between biological molecules based on biological networks has been widely used in many types of research, such as searching for gene sequencing of a disease [27] and predicting drug target interactions. Some of them have achieved good prediction results and good performance. Xiao et al. [35] proposed the PLPIHS method (Figure 6) to predict lncRNA–protein interactions using HeteSim scores and they used a path metric to calculate the interrelationship between nodes in heterogeneous networks. Zeng et al. [105] inferred the association between heterogeneous nodes by means of uniform and symmetric metrics of random paths, regardless of whether they are the same or different types according to the score. Because the relevance path captures the semantic information and also also restricts the wandering path, the score depends on the similarity measure of the path. A heterogeneous network is first constructed with an lncRNA–lncRNA similarity network, which uses the Pearson’s correlation coefficient between the expression profiles of each pair of lncRNAs to calculate the lncRNA–protein association network downloaded from GENCODE Release 24 [106] and a PPI network obtained from the STRING v10.0 database [107]. Then, they used the HeteSim to measure the degree of interaction of each lncRNA–protein in the network and showed it in fractions. The SVM classifier is built on the basis of the scores of different paths.

LOOCV is carried out to evaluate the performance of PLPIHS [108]. The results show that the AUC of PLPIHS for the network cutoff value of 0.3 is 96.8%, which is higher than LPIHN. Similarly, PLPIHS outperforms other methods in the 0.5 network and 0.9 network as well. A total of 2000 lncRNA–protein associations from positive samples of the 0.9 network and 2000 interactions from the remaining negative samples of the 0.3 network are randomly selected to construct an independent test set to further conduct the performance evaluation. Using this independent test set, PLPIHS achieves an AUC value of 0.879.

## 5. Results of Comparisons of the Network-Based Models for Predicting LncRNA–Protein Interactions

To compare the network-based methods, the fusion of heterogeneous data and performance evaluation were analyzed. All of the above-described methods used LOOCV to validate their respective performances. The test results of the network-based methods are shown in Table 5.

Yang et al. [33] proposed that the relevance path is the same as fusing multiple PPSNs. They extracted MALAT1 and AK0951949, respectively, with all 99 proteins as two experimental datasets. Known interactions data between two lncRNAs and their protein chaperones are considered as positive samples, while negative samples are new pairs of lncRNA–protein interactions that have not been experimentally verified. From the ROC curves of the prediction results, the AUC is 0.955 for MALAT1 with all 99 proteins and 0.973 for AK0951949 with all 99 proteins.

LPBNI obtained 4870 lncRNA–protein interactions data from NPInter 2.0. The method used the propagation matrix and the lncRNA–protein interaction networks to set the test sample. First, the test sample is set according to the interaction pair of each lncRNA–protein in the adjacent matrix, leaving a node and setting one at the zero corresponding value of the adjacent matrix. In this process, some nodes will be deleted during the evaluation process. Considering that these nodes do not have more than two connection nodes, it is considered that there is no information dissemination between them. Compared with random walk with restart, it is clear that LPBNI showed the highest true positive rate in each false positive rate, and the AUC value was 0.878.

PLPIHS selected data samples in different cutoff values of networks and obtained 2000 positive samples from 0.9 network and randomly selected 2000 negative samples from 0.3 network. PLPIHS calculated the AUC in different network cutoff values (0.3 and 0.9), where that for the 0.3 network was 0.968, which was higher than the value calculated by LPIHN. To verify that PLPIHS has better performance, the authors select the same number of positive and negative samples from different cutoff values of the network, respectively, and use this random selection to construct independent test datasets. Compared with the values generated by LPIHN, the AUC value of PLPIHS was 0.879. The accuracy, sensitivity, precision, Matthew’s correlation coefficient, and F1-Score were also chosen as measurements to evaluate performance.

Fusing multiple PPSNs to effectively predict lncRNA–protein interactions was from the perspective of a fusion protein. The best relevance path was lncRNA–protein–protein according to HeteSim. The fusion matrix is an effective means for users to get more reliable and richer information matrix or network. The best AUC value was 0.9068 with Go+Pfam, Go+String, and Pfam+String. The AUC values of the 15 settings implemented in the paper by Zheng et al. [32] are shown in Table 5, which included using only one similarity matrix, fusing two similarity matrices, fusing three similarity matrices, and fusing all four similarity matrices.

In the LPIHN model, the determination of test datasets is consistent with other interaction prediction methods, leaving a cross-validation method. This model used not only LOOCV but also precision versus recall curves and fold enrichment to measure the performance, whereas the average fold enrichment of all test data was used for assessment. The LOOCV results showed that LPIHN obtained an AUC of 0.96. When more attention was paid to the predicted first 4870 lncRNA–protein interactions, 802 of the predicted LPIHN interactions were within this ranking.

**Figure 7:**
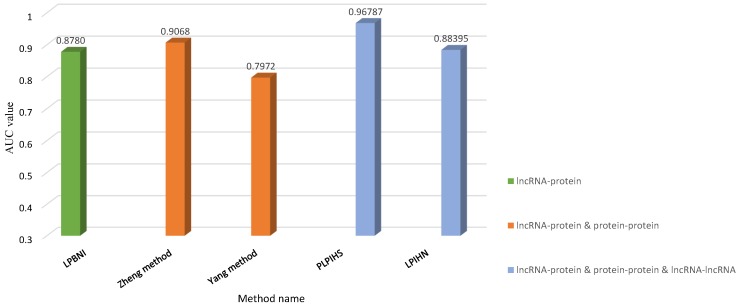
The AUC value of five methods under at three different levels of heterogeneous networks. Different colors represent different network cases, and the same color bar graphs represent the verification results on the same set of data.

To better understand the performance of network-based computational models to predict lncRNA–protein interactions, we divided the heterogeneous network into three cases according to the source of components: (1) only the lncRNA–protein interaction network; (2) the network combining the interactions of lncRNA–protein and protein–protein; and (3) the network with the integration of the interactions of lncRNA–protein, protein–protein and lncRNA–lncRNA. For each case, different methods were validated with the same set of test datasets, and the performances are compared by AUC in Figure 7. LPBNI (green) used leave-one-out cross validation on 4796-lncRNA–protein interaction network. The method proposed by Yang et al. [33] and method (orange) by Zheng et al. [32] used leave-one-out cross validation on 4467 lncRNA–protein interaction networks. The remaining two methods (blue) used leave-one-out cross validation on the dataset which 2000 lncRNA–protein interactions from network of PLPIHS with cutoff of 0.9 were extracted as positive samples, 2000 negative samples were randomly selected in 0.3 network. The gold set containing 185 lncRNA–protein interactions downloaded from NPInter database. In Figure 7, different colors represent different network types, and the same color bar graphs represent the verification results under the same set of data. In Figure 7, the performance of the method is better when the heterogeneous network is composed by more sources. When heterogeneous networks are constructed by the same sources, the performance will be better for the heterogeneous networks constructed by weighted networks. (The implication of more data here can be illustrated by the interactions of lncRNA–lncRNA. The interactions of lncRNA–lncRNA can be considered from many perspectives. It can be calculated from expression profile data, sequence alignment or experiment.) For example, the method proposed by Yang et al. and method (orange) by Zheng et al. both integrated the interactions of lncRNA–protein and protein–protein to construct a heterogeneous network, and both methods were based on the relevant path of HeteSim random walk in the heterogeneous network. However, for protein–protein interaction networks construction, Yang et al. only considered the protein–protein interactions from STRING database. Zheng et al. considered not only the protein–protein interactions from STRING database, but also the sequence similarity, functional annotation semantic similarity and protein domain similarity protein–protein interactions constructed based on. The method (orange) by Zheng et al. with AUC 0.9068 is better than the method proposed by Yang et al. with AUC 0.7972.

## 6. Discussion

Prediction of the interactions between lncRNAs and proteins is a very important step for research about lncRNAs. Based on the results of lncRNA–protein interactions, the functions as well as the associated diseases of lncRNAs can be inferred. The lncRNA–protein interaction is a very significant molecular mechanism. Computational approaches to predict lncRNA–protein interactions can be grouped into two broad categories. The first category is based on intrinsic features of the lncRNAs and proteins, including the sequence, structural information, and physicochemical property. The second category is based on the fusion of heterogeneous data to construct a network.

Whereas the sequence-based methods only consider the properties of the RNA and neglect the internal relationship between the lncRNAs and proteins, the network-based methods have paid more attention to this kind of internal relationship. The main advantage of a network-based computational model is that it can predict lncRNA–protein interactions with heterogeneous data. Predictions using the intrinsic features of sequences alone may lead to more false-positive interaction pairs than that obtained using a network-based method. Unavoidably, the network-based computational model can have some disadvantages. The prediction of the network-based computational model can be affected when it is carried out in the case of finite interactions. When there are no interaction data, the network-based computational model cannot be used to predict interactions.

New lncRNA–protein interactions are predicted more effectively by using several kinds of heterogeneous data sources. As the study of proteins becomes ever more comprehensive, the proposed effective computational models for predicting lncRNA–protein interactions from heterogeneous biological data can benefit our understanding of more comprehensive annotations for lncRNAs.

Currently, there is very limited information on the interaction between lncRNAs and proteins, but computational methods can provide us with a large number of interaction pairs that can be further regarded as valuable material for the inference of lncRNA functions. First, a great deal of lncRNA–protein interactions can be provided by computational models based on intrinsic features. Second, since predictions using the intrinsic features of sequences alone may lead to some false-positive interaction pairs, computational models based on biological networks can be chosen to obtain more reliable predictions. In the future, a deep-learning-based framework can be considered to optimize the sequence-based and network-based computational models. Hopefully, long-short-term memory models can be employed to build a more advanced framework to build classifiers and achieve a more reliable classification model. We also can integrate machine learning with ab initio computing and network representation learning methods, and apply them to the prediction model of relationships between biological macromolecules. The interactions between lncRNAs and other molecules may enrich the functional annotations of lncRNAs. First, researchers can extract the characteristics of the molecules themselves by machine learning algorithm, and then they can use the appropriate algorithm in network representation learning to represent the feature vectors of relationships between nodes in heterogeneous networks. In this way, researchers can not only understand the internal features of molecules, but also not ignore the hidden topological information between molecules. This will overcome the weakness of most current research methods which only consider ab initio prediction or network-based methods.

## Figures and Tables

**Table 1:** Description of lncRNA relevant databases.

Database	Description	Availability
ncRNA database (Especially lncRNAs):		
NONCODE [54]	Comprehensive knowledge database of non-coding RNAs, including lncRNAs from 17 species, and predicted/validated lncRNA–disease relationships.	http://www.noncode.org
MNDR [55]	Database of ncRNA–disease associations in mammals.	http://www.rna-society.org/mndr
deepBase [56]	Database for identification, expression, evolution and function of small RNAs, lncRNAs and circular RNAs from deep-sequencing data.	http://rna.sysu.edu.cn/deepBase
NRED [57]	Database integrating annotated human and mouse ncRNA expression data from various resources.	http://nred.matticklab.com
ChIPBase [58]	Database on the transcriptional regulation of ncRNAs based on ChIP-sequencing data.	http://rna.sysu.edu.cn/chipbase
SomamiR [59]	Cancer somatic mutations with altering microRNA–ceRNA interactions.	http://compbio.uthsc.edu/SomamiR
LncRNA2Function [60]	Functional annotations and expression profiles (RNAseq) of human lncRNAs.	http://mlg.hit.edu.cn/lncrna2function
LincSNP [61]	A database containing human lncRNAs information about linking disease related SNPs.	http://bioinfo.hrbmu.edu.cn/LincSNP
LncRNA-SNP [62]	A database of SNPs in lncRNAs and their predicted effects in human and mouse.	http://bioinfo.life.hust.edu.cn/lncRNASNP
LNCipedia [63]	A database for annotated human lncRNA transcript sequences and structures.	http://www.lncipedia.org
ALDB [64]	A farm livestock lncRNA database.	http://res.xaut.edu.cn/aldb/index.jsp
lncRNAtor [65]	A database for functional investigation of lncRNAs that encompasses annotation, sequence analysis, gene expression, protein binding and phylogenetic conservation.	http://lncrnator.ewha.ac.kr
Co-LncRNA [66]	A web-sever containing effects of lncRNAs in GO functions and KEGG pathways based on co-expressed genes.	http://www.bio-bigdata.com/Co-LncRNA
Lnc2Cancer [17]	A database for experimentally validated associations between lncRNAs and cancers.	http://www.bio-bigdata.net/lnc2cancer
LncRNADisease [16]	A database for experimentally validated lncRNA-associated diseases.	http://www.cuilab.cn/lncrnadisease
lncRNAMap [67]	A map of putative regulatory functions in the long non-coding transcriptome.	http://lncRNAMap.mbc.nctu.edu.tw/
TANRIC [34]	A web-resource for interactive exploration of lncRNAs in cancer.	http://ibl.mdanderson.org/tanric/_design/basic/index.html
LncRNA ontology [64]	A web-resource for inferring lncRNA functions based on chroma-tin states and expression patterns.	http://www.bio-bigdata.com/lncrnaontology/
LNCediting [68]	A database for functional effects of RNA editing in lncRNAs.	http://bioinfo.life.hust.edu.cn/LNCediting/
LncBase [69]	A database of interactions between miRNAs and lncRNAs.	http://www.microrna.gr/LncBase
TF2LncRNA [70]	A Web-resource for the identification of common transcription factors for a list of lncRNA genes.	http://mlg.hit.edu.cn/tf2lncrna
LncSubpathway [71]	A web server for the identification of dysfunctional subpathways associated with risk lncRNAs.	http://www.bio-bigdata.com/lncSubpathway/
LncRNA2Target [72]	A database of differentially expressed genes after lncRNA knock-down or overexpression.	http://lncrna2target.org
LncReg [73]	A reference resource for lncRNA-associated regulatory networks.	http://bioinformatics.ustc.edu.cn/lncreg/
lncRNAdb [74]	An annotation database of eukaryotic lncRNAs.	http://www.lncrnadb.org/
Database information on proteins or microRNAs that may be associated with lncRNAs:		
NPInter [75]	Database of noncoding RNA-associated interactions.	http://www.bioinfo.org/NPInter
PRIDB [76]	Comprehensive database of RNA–protein interfaces extracted from complexes in the PDB.	http://bindr.gdcb.iastate.edu/PRIDB
PDB [77]	A database of experimentally determined three-dimensional structures of proteins, nucleic acids and other biomolecules.	http://www.rcsb.org/
StarBase v 2.0 [78]	A database of experimentally supported interactions from RBPs, mRNAs, miRNAs, RNAs, proteins and so on.	http://starbase.sysu.edu.cn/
Nucleic acid database (NDB) [79]	A database about three-dimensional nucleic acid structures and their complexes, geometric data, structure information.	http://ndbserver.rutgers.edu/

**Table 2:** Details of interactions between biomolecules and the research of lncRNA functions.

Name	Samples	Interactions	Source
LncRNA–Disease	804 × 288	1454	LncRNADisease [16], Lnc2Cancer [17]
LncRNA–LncRNA	1114 × 1114	1,179,256	LFSCM [80]
LncRNA–microRNA	1127 × 277	10,198	StarBase v2.0 [78]
LncRNA–Gene	240 × 15,527	6186	LncRNA2Target [72]
LncRNA–GO	240 × 6428	3094	GeneRIF [81]
MicroRNA–MicroRNA	271 × 271	24,062	Zhong et al. [82]
MicroRNA–Disease	1080 × 592	11,835	HMDD [83], miR2Disease [84], miRCancer [85]
MicroRNA–Gene	495 × 15,527	135,852	miRTarBase [86]
MicroRNA–Target	495 × 15,527	135,852	miRTarBase [86]
Gene–Gene	16,785 × 16,785	1,515,370	Yao et al. [49]
Gene–Metabolite	12,342 × 3278	192,763	Yao et al. [49]
Metabolite–Metabolite	3764 × 3764	74,667	Yao et al. [49]
Gene–GO	15,527 × 6428	1,191,503	GO Annotation [87]
Gene–Disease	1715 × 1886	2603	DisGeNET [88]
Gene–Drug	155,275 × 8283	3760	DrugBank [89]
Metabolite–Disease	388 × 149	664	HMDB [90]
Drug–Disease	15,527 × 412	115,317	CTD [91]
Drug–Drug	8283 × 8283	453,436	DrugBank [89]
Drug–Side-effects	1430 × 5880	140,064	SIDER [92]
Disease–Disease	5080 × 5080	20,280,092	Yao et al. [49]

**Table 3:** The comparison of each method by analyzing the differences in intrinsic features and classifiers.

		CatRAPID [26]	RPISeq [24]	De novo [25]	LncPro [5]	RPI-Pred [27]	rpiCOOL [28]	IPMiner [29]	lncADeep [30]
**Feature**	RNA Sequence		√	√	√	√	√	√	
	Protein Sequence		√	√	√	√	√	√	
	3D Structure(RNA)					√			
	3D Structure (protein)					√			
	The secondary structure (RNA)	√			√				
	The secondary structure(protein)				√				
	Hydrogen-Bonding Propensities	√			√				
	van der Waals’ Propensities	√			√				
**Classifier**	Random Forest		√				√	√	
	Naive Bayesian			√					
	Extended NB			√					
	SVM		√			√			
	Fisher’s linear				√				
	automatic encoder							√	
	deep neural network								√
	*p*-values	√							√
Web server or offline package		√	√		√	√	√	√	√

^1^http://s.tartaglialab.com/page/catrapid_group (web server); ^2^ http://pridb.gdcb.iastate.edu/RPISeq (web server); ^3^ http://bioinfo.bjmu.edu.cn/lncpro/ (offline package and web server); ^4^ http://ctsb.is.wfubmc.edu/projects/rpi-pred (web server); ^5^ http://biocool.ir/softs/rpicool.html (offline package); ^6^ https://github.com/xypan1232/IPMiner (offline package); ^7^ https://github.com/cyang235/LncADeep (offline package).

**Table 4:** Differences in each network-based methods.

Method	Dataset		Algorithm	AUC
LPBNI [31]	LPI	4870 lncRNA–protein interactions from NPInter database (2380 lncRNAs and 106 proteins)	Bipartite Network	0.8780
	PPI	×		
	LLI	×		
Yang et al. [33]	LPI	4883 lncRNA–protein interactions from NPInter database (1116 lncRNAs and 99 proteins)	A random walk model HeteSim	0.7972
	PPI	1608 protein–protein interactions from STRING database		
	LLI	×		
LPIHN [34]	LPI	10232 lncRNA–protein interactions from NPInter database (1113 lncRNAs and 99 proteins)	Random Walk with Restart	0.8839
	PPI	804 protein–protein interactions from STRING database		
	LLI	lncRNA expression similarity from NONCODE 4.0 database (1113 lncRNA expression profiles)		
Zheng et al. [32]	LPI	4467 lncRNA–protein interactions from NPInter database (1050 lncRNAs and 84 proteins)	SNF; A random walk model HeteSim	0.9068
	PPI	Sequence similarity from UniProt database; Functional annotation similarity from GO database; Protein domain similarity from Pfam database; STRING similarity from STRING database;		
	LLI	×		
PLPIHS [35]	LPI	lncRNA–protein interactions from GENCODE Release 24 (15941 lncRNAs and 20284 proteins) Co-expression data from COXPRESdb; Co-expression data from ArrayExpress and GEO; lncRNA–protein interactions from NPInter database;	SVM; A random walk model HeteSim	**0.9678**
	PPI	Protein–protein interactions from STRING database		
	LLI	lncRNA co-expression similarity from NONCODE database (lncRNA expression profiles)		

**Bold** representation performs best in AUC values and we found that the performance of the method is better when the heterogeneous network is composed by more sources. When heterogeneous networks are constructed by the same sources, the performance will be better for the heterogeneous networks constructed by weighted networks. ^1^ https://github.com/USTC-HIlab/LPBNI (offline package); ^2^ https://github.com/cyang235/LncADeep (offline package); ^3^ lncRNA–protein interactions; ^4^ protein–protein interactions; ^5^ lncRNA–lncRNA interactions; ^6^ A relevance search based on random walk in heterogeneous network to evaluate the relevance between a pair of lncRNA and protein, and a large relevance score means a high possibility that the lncRNA and protein interacts [94]. ^7^ Similarity Network Fusion: It is a nonlinear message-passing based method that iteratively updates each network and makes it more and more similar to the other [95].

**Table 5:** Differences in evaluation measures by the network-based methods.

Method	Measure for the Evaluation			Test Dataset	Measurement or Illustration							
	LOOCV	Precision Versus	Fold Enrichment		AUC	SPE	ACC	PRE	MCC	REC	F1-Score	SEN
LPBNI [31]	√		√	4870 lncRNA–protein interactions from NPInter v2.0	0.878	0.99	0.873	0.852	0.449	−	−	0.288
						0.95	0.880	0.681	0.534			0.532
Zheng et al. [32]	√			4467 lncRPIs, including 1050 lncRNAs and 84 proteins	AUC values of 15 settings: Seqs-0.8565, Pfam-0.8459, GO-0.8584, STRING-0.7972; Seqs+Pfam-0.8689, Seqs+GO-0.8626, Seqs+STRING-0.8762, Pfam+GO-0.8677, Pfam+STRING-0.8977, and GO+STRING-0.8814; Seqs+Pfam+GO-0.8704, Seqs+Pfam+STRING-0.9023, Seqs+GO+STRING-0.8904, Pfam+GO+STRING-0.9066; Seqs+Pfam+GO+STRING-0.9068.							
Yang et al. [33]	√			MALAT1 with all 99 proteins	0.955	−	−	−	−	−	−	−
				AK0951949 with all 99 proteins	0.973							
LPIHN [34]	√	√	√	The test dataset is the interaction of each known lncRNA–protein, and the rest is used as training dataset.	0.96	√	√	√	√	√		√
PLPIHS [35]	√			The remaining positive samples found in the 0.9 network had 2000 lncRNA–protein interactions and the same number of negative interactions in the 0.3 network	0.879	−	√	√	√		√	√

LOOCV, leave-one-out cross validation; AUC, area under the curve; SPE, specificity; ACC, accuracy; PRE, precision; MCC, Matthew’s correlation coefficient; REC, recall; SEN, sensitivity; OMIM, Online Mendelian Inheritance in Man compendium.
